# Serial Femtosecond Zero Dose Crystallography Captures a Water‐Free Distal Heme Site in a Dye‐Decolorising Peroxidase to Reveal a Catalytic Role for an Arginine in Fe^IV^=O Formation

**DOI:** 10.1002/anie.202008622

**Published:** 2020-09-23

**Authors:** Marina Lučić, Dimitri A. Svistunenko, Michael T. Wilson, Amanda K. Chaplin, Bradley Davy, Ali Ebrahim, Danny Axford, Takehiko Tosha, Hiroshi Sugimoto, Shigeki Owada, Florian S. N. Dworkowski, Ivo Tews, Robin L. Owen, Michael A. Hough, Jonathan A. R. Worrall

**Affiliations:** ^1^ School of Life Sciences University of Essex Wivenhoe Park Colchester Essex CO4 3SQ UK; ^2^ Diamond Light Source Harwell Science and Innovation Campus Didcot Oxfordshire OX11 0DE UK; ^3^ RIKEN Spring-8 Center 1-1-1 Kouto Sayo Hyogo 679-5148 Japan; ^4^ Japan Synchrotron Radiation Research Institute 1-1-1 Kouto Sayo Hyogo 679-5198 Japan; ^5^ Swiss Light Source Paul Scherrer Institute 5232 Villigen PSI Switzerland; ^6^ Biological Sciences Institute for Life Sciences University of Southampton University Road Southampton SO17 1BJ UK

**Keywords:** arginine, bioinorganic, heme proteins, peroxidase, X-ray serial femtosecond crystallography

## Abstract

Obtaining structures of intact redox states of metal centers derived from zero dose X‐ray crystallography can advance our mechanistic understanding of metalloenzymes. In dye‐decolorising heme peroxidases (DyPs), controversy exists regarding the mechanistic role of the distal heme residues aspartate and arginine in the heterolysis of peroxide to form the catalytic intermediate compound I (Fe^IV^=O and a porphyrin cation radical). Using serial femtosecond X‐ray crystallography (SFX), we have determined the pristine structures of the Fe^III^ and Fe^IV^=O redox states of a B‐type DyP. These structures reveal a water‐free distal heme site that, together with the presence of an asparagine, imply the use of the distal arginine as a catalytic base. A combination of mutagenesis and kinetic studies corroborate such a role. Our SFX approach thus provides unique insight into how the distal heme site of DyPs can be tuned to select aspartate or arginine for the rate enhancement of peroxide heterolysis.

## Graphical abstract

Femtosecond X‐ray crystallography reveals the pristine structures of the Fe^III^ and Fe^IV^=O redox states of a B‐type dye‐decolorising heme peroxidase. These structures reveal a water‐free distal heme site, which together with the presence of an asparagine, imply the operation of the distal arginine as a catalytic base in the heterolysis of peroxide.

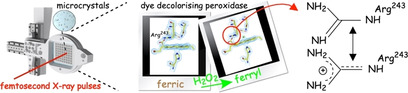

## Introduction

Capturing structures of intact redox states in metalloenzymes, whilst challenging, is critical for assigning the chemistry of the metal in the catalytic cycle. Substantial efforts have been made to define the chemical nature and thus reactivity of the high‐valent Fe^IV^‐O intermediates in iron‐containing enzymes, commonly referred to as ferryl.^[1]^ Many heme enzymes use ferryl species as part of their catalytic cycle, where heme peroxidases are a prominent example.^[2]^ Heme peroxidases react with peroxide by removing one electron from the heme‐Fe^III^ and a second from the porphyrin to generate a porphyrin π‐cation radical; in cytochrome c peroxidase (CcP) an electron is taken from a tryptophan to form a tryptophanyl radical.^[2a]^ The two‐electron oxidized species is commonly referred to as compound I.[^2b^, ^2c^]

X‐ray and resonance Raman approaches have focused on measuring the Fe^IV^−O bond length and strength, which for Fe^IV^=O is shorter and stronger than Fe^IV^−OH species.^[2d]^ However, a major complication has been the sensitivity of the heme‐Fe to reduction caused by the incident X‐rays and lasers.^[2d]^ X‐rays interact with protein crystals to generate large numbers of solvated photoelectrons, and the effects manifest first in electron rich sites such as metals to rapidly change, for example, the electronic state of the heme‐Fe and with it the structure of the active site. Alternative approaches have been sought to obtain intact (undamaged) or near‐intact structural data of radiation‐sensitive Fe^III^ and Fe^IV^ heme species. These include low‐dose composite (i.e. multi‐crystal) X‐ray structures coupled with single crystal spectroscopy (near‐intact),^[3]^ as well as neutron diffraction^[4]^ and structures determined by X‐ray free electron lasers (XFELs, zero dose)^[5]^ to obtain fully intact structures.

Serial femtosecond crystallography (SFX) using XFELs allows diffraction data of a crystal to be collected with femtosecond exposure times that can outrun the effects on the structure of X‐ray induced photoreduction/radiation damage. Crystal structures determined in this way preserve the redox state of the metal site and can be considered pristine (i.e. free of radiation‐induced damage).[^5a^, ^6^] As the XFEL pulse destroys the crystal, a new crystal is required for each pulse, explaining the need for serial methods that use many thousands of microcrystals. Efficient sample delivery systems are therefore essential for SFX experiments.

We previously reported a SFX approach that used microcrystals of metalloenzymes (≤50 μm), where a silicon fixed‐target chip containing 25 600 apertures was used to trap crystals.[^5b^, ^7^] An advantage of this delivery system is that SFX data may be collected at ambient temperature, as opposed to experiments at cryogenic temperatures (100 K) where structural states are frozen out, and chemistry is slowed.^[8]^ Ambient temperature crystallography is particularly challenging due to a greatly increased susceptibility to the observed effects of radiation damage in comparison to that which occurs at 100 K.^[9]^ The application of our chip based approach to determine pristine structures of heme‐Fe redox states in peroxidases under ambient temperatures is therefore attractive,[^5b^, ^7^] but requires the ferryl species to be stable throughout the duration of the SFX experiment (typically <20 min using our system).

Dye‐decolorising peroxidases (DyPs) are the most recent member of the peroxidase family to be discovered.^[10]^ They differ significantly in protein fold to their nonmammalian counterparts for example, CcP and horseradish peroxidase (HRP).^[11]^ Three DyP subfamilies (types A, B, C/D) exist^[12]^ and all react with peroxide to form a compound I species carrying a porphyrin π‐cation radical. A distinguishing feature of DyPs is the absence of a distal heme His,^[11]^ which in nonmammalian peroxidases plays a prominent role in enhancing the rate of compound I formation.^[13]^ The distal His acts together with a hydrogen‐bonded (H‐bonded) H_2_O molecule to facilitate proton movement of the Fe‐linked peroxide O atom to the distal O atom promoting heterolytic cleavage of the O−O bond to form compound I and the subsequent release of a H_2_O molecule.[^5a^, ^14^] In contrast, a distal Asp is found in DyPs.

Validated redox‐state structures for DyPs have only been reported for two A‐type homologues from *Streptomyces lividans*,[^3d^, ^3e^, ^5b^, ^15^] providing much needed structural clarity into deciphering the mechanistic features associated with compound I formation in the DyP family.^[3e]^ Significantly, a distal H_2_O‐Asp unit (analogous to the H_2_O‐His unit in nonmammalian peroxidases) was found and experimentally demonstrated to facilitate rapid proton transfer for compound I formation.^[3e]^ However, its presence alone is not guaranteed to enhance compound I formation, as a subtle perturbation in the positioning of the H_2_O‐Asp unit drastically suppresses reactivity with peroxide, as revealed in one of the A‐type homologs.^[3e]^ Thus, validating the redox state of the Fe^III^ heme structure in these DyP homologues has proved critical for elucidating a H_2_O‐Asp mechanism, as reduction of the Fe^III^‐heme to the Fe^II^ state alters the distal H‐bonded H_2_O network and would have led to mechanistic misinterpretation.^[15]^

In nonmammalian peroxidases a distal heme Arg is present that is indirectly involved in compound I formation.[^2c^, ^13c^, ^16^] A distal heme Arg is conserved amongst DyP members, however, unlike in nonmammalian peroxidases experimental evidence for some DyPs suggests that the Arg plays a direct role in the heterolytic cleavage of the bound peroxide to form compound I,^[17]^ whereas in others the Asp is the proton acceptor/donor.[^3e^, ^11^, ^18^] Thus an outstanding question is how the heme site in DyPs can modulate the distal Asp/Arg to facilitate proton transfer and the rate enhancement of O‐O heterolysis? Here, through determining zero dose SFX structures of the Fe^III^ and Fe^IV^ redox states of a B‐type DyP (DtpB) from *S. lividans*, together with spectroscopic, kinetic and mutagenesis data we reveal how Arg is “selected” by certain DyPs to act as a base catalyst for compound I formation.

## Results and Discussion

**Figure 1:**
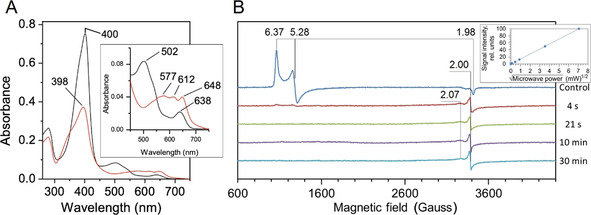
A) Electronic absorption spectrum of Fe^III^‐DtpB (black) and compound I (red) generated by addition of one molar equivalent H_2_O_2_ (pH 5.0). Wavelength (nm) absorbance maxima are indicated. Inset: magnified Q‐band region. B) X‐band EPR spectra (10 K) of Fe^III^‐DtpB (control) and spectra of four samples frozen at variable times after addition of one molar equivalent H_2_O_2_. The principal *g*‐values in each spectrum are indicated with the zero field splitting rhombicity parameter E/D=0.023. Inset: dependence of the *g*=2 compound I signal on microwave power.

**Figure 2:**
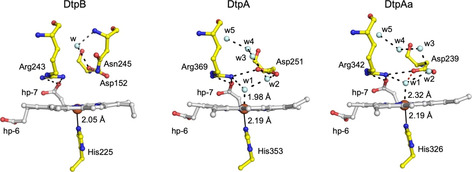
The SFX heme site structure of Fe^III^‐DtpB and comparison with the redox state validated Fe^III^‐DtpA^[3d]^ and Fe^III^‐DtpAa^[5b]^ structures. Water molecules (w) are depicted as cyan spheres, hp refers to heme propionate groups and H‐bond interactions are shown as dashed lines. In DtpAa the red dashed line indicates an additional H‐bond to w1 that is absent in DtpA—a feature that influences compound I formation.^[3e]^

Three DyPs have been identified in *S. lividans*; two A‐types (DtpA and DtpAa) and one B‐type (DtpB).^[19]^ Herein we focus on DtpB, which has an electronic absorbance spectrum typical for a high‐spin (HS) Fe^III^‐heme (Figure 1 A). Addition of one molar equivalent of H_2_O_2_ to Fe^III^‐DtpB leads to rapid formation of a green colored species, associated with a 2 nm blue wavelength shift and a flattening of the Soret band in the electronic absorbance spectrum (Figure 1 A). These changes are typical for a compound I species carrying a porphyrin π‐cation radical. No further change in the electronic absorbance spectrum following addition of H_2_O_2_ to DtpB is observed for >1 h at room temperature (RT), indicating DtpB forms a highly stable compound I species. Low temperature (10 K) EPR spectroscopy of Fe^III^‐DtpB reveals spectral features typical of a HS Fe^III^‐heme (Figure 1 B). On addition of one molar equivalent of H_2_O_2_ to Fe^III^‐DtpB a series of samples were frozen at variable time points and the EPR spectrum recorded. After 4 s the HS Fe^III^‐heme signal has all but disappeared (<1 % of the initial concentration) with a new signal appearing at the *g*=2 position and a possibly related component at *g*=2.07. The *g*=2 signal has a strongly asymmetric line‐shape that does not change in intensity over a 30 min period and shows no saturation with microwave power (inset Figure 1 B). Together these features are typical of a compound I species carrying a porphyrin π‐cation radical. The kinetics of compound I formation in DtpB were monitored using stopped‐flow absorption spectroscopy at pH 5.0. A single spectral transition on mixing Fe^III^‐DtpB with H_2_O_2_ was observed consistent with a transition from a Fe^III^‐heme to compound I. A linear dependence of pseudo first‐order rate constants obtained from the global fitting of the spectral data with increasing [H_2_O_2_] was observed enabling a second‐order rate constant (*k*_1_) of 2.9±0.1×10^5^ M^−1^ s^−1^ (25 °C) to be determined (Supporting Information, Figure S1A). Compound I formation in DtpA is facilitated through the distal H_2_O‐Asp unit (w2 in Figure 2) with *k*_1_=1×10^7^ M^−1^ s^−1^, some 2‐orders of magnitude greater than for DtpB.[^3e^, ^20^] However, DtpA compound I has a *t*_1/2_≈2.5 min,^[20]^ whereas for DtpB the life‐time is considerably longer (Figure 1 B), thus permitting structural investigation of compound I using our chip‐based SFX approach.

Addition of H_2_O_2_ to DtpB microcrystals for SFX experiments resulted in an instant color change from brown to green, consistent with compound I formation. The heme redox state in the crystals was corroborated through analysis of larger DtpB crystals suitable to obtain electronic absorbance spectra at 100 K.^[21]^ Identical electronic absorbance bands to those for Fe^III^‐DtpB and compound I in solution were observed in the crystals (Figure S2). SFX data for Fe^III^ and compound I microcrystals of DtpB were collected using the SACLA XFEL beamline BL2 EH3. Data collection, processing and refinement statistics are reported in Tables S1 and S2 (Supporting Information).

The RT SFX structures of Fe^III^ and compound I DtpB were determined to 1.85 Å and 1.75 Å resolution, respectively, and reveal a typical DyP two‐domain ferredoxin fold (Figure S3), with six monomers in the crystallographic asymmetric unit. Inspection of the Fe^III^‐DtpB distal heme site (Figure 2) reveals several differences when compared with the only other redox state validated Fe^III^ structures of the DyP family, DtpA^[3d]^ and DtpAa[^3e^, ^5b^] (Figure 2). The heme‐Fe in DtpB is penta‐coordinate and sits out of the porphyrin plane towards the proximal His225 ligand. Such a distortion results in a short Fe^III^‐N^δ^‐His bond length (2.05±0.13 Å in monomer A, Table S3) compared to 2.19 Å reported in Fe^III^‐DtpA and Fe^III^‐DtpAa.[^3d^, ^3e^, ^5b^] The N^*ϵ*^ of His225 is H‐bonded to the O^δ2^ atom of Asp287, an interaction that imparts significant imidazolate character in histidine ligated heme peroxidases resulting in increased electron‐donating ability.^[2c]^ Fe^III^‐DtpA and Fe^III^‐DtpAa have a hexa‐coordination heme geometry with a H_2_O molecule occupying the distal coordination position, which forms the origin for an extensive H‐bonded H_2_O network connecting the distal heme‐Fe to bulk solvent.^[3d]^ This H_2_O network communicates with the distal Asp (Figure 2), which in DtpA is optimized for rapid proton movement and compound I formation.^[3e]^ The distal heme site in the immediate vicinity of the Asp/Arg couple of Fe^III^‐DtpB is thus “dry”, which has the effect of isolating Asp152 from communication with the heme. Moreover, once peroxide is bound the absence of the H_2_O‐Asp unit makes the distance directly from an O^δ^ atom of Asp152 to the Fe^III^‐HOOH complex ≈3.5 Å which would not be optimal for either direct proton abstraction or delivery to compound 0 (Fe^III^‐OOH^−^).

The absence of a distal heme‐Fe H_2_O chain in Fe^III^‐DtpB coincides with the protrusion into the distal site of Asn245, with its side chain amide occupying the spatial position where a H_2_O molecule (w2, Figure 2) is located and H‐bonded to the distal Asp in the Fe^III^‐DtpA and Fe^III^‐DtpAa structures. This H_2_O‐Asp unit facilitates rapid formation of compound I in DtpA.^[3e]^ Asn245 therefore adds a steric impediment to the distal site in DtpB, and as a consequence prevents the formation of the heme‐Fe H_2_O networks observed in DtpA and DtpAa.[^3d^, ^3e^, ^5b^] The amide of Asn245 is orientated to facilitate H‐bond formation with both the O^δ^ atoms of Asp152, albeit with distances >3 Å. In common with DtpA and DtpAa, both the N^η^ atoms of Arg243 are H‐bonded with the O1A atom of the inwardly pointing heme propionate‐7 (Figure 2). Solvent access to the heme is through a surface entrance adjacent to heme propionate‐6. The larger diameter of the surface entrance in DtpB greatly increases the accessible solvent area (ASA) of the heme in Fe^III^‐DtpB (37.4 Å^2^) compared to Fe^III^‐DtpA (11.2 Å^2^) and Fe^III^‐DtpAa (11.8 Å^2^) where the heme is more solvent insulated (Figure S4). To seek further structural confirmation for a “dry” distal site in Fe^III^‐DtpB a composite X‐ray structure of Fe^III^‐DtpB at 100 K was determined (Tables S1 and S2). Consistent with the SFX structure, the spectroscopically‐validated 100 K composite structure also revealed a penta‐coordinate and out of plane heme‐Fe and importantly confirmed the absence of a H_2_O‐Asp unit (Figure S5).

**Figure 3:**
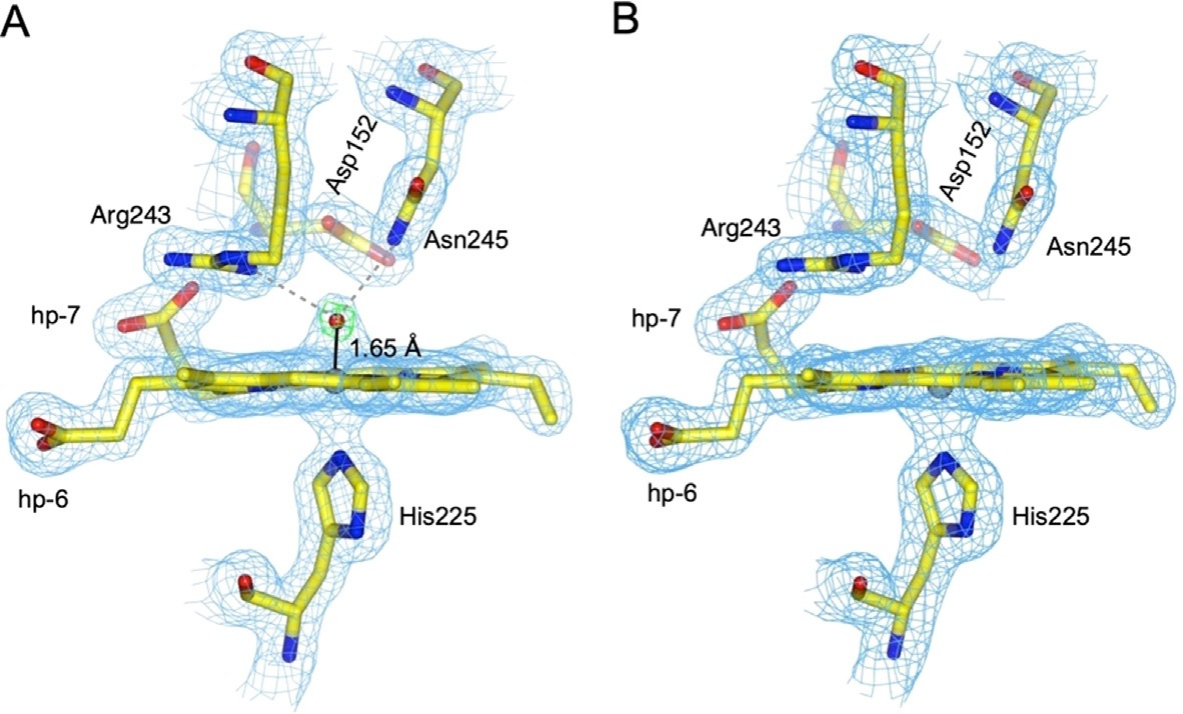
Heme sites of the A) Fe^IV^=O and B) Fe^III^ states of DtpB determined by SFX at RT. 2 *F*_o_−*F*_c_ electron density maps (blue) contoured at 1.4 σ for the Fe^IV^=O (A) and Fe^III^ (B) oxidation states for monomer A. In (A) the *F_o_‐F_c_* omit map (green) is also shown, contoured at ±10 σ. This was calculated after refinement, omitting the oxygen atom (red sphere). The Fe to O coordination bond is shown as a solid black line and H‐bond interactions involving the O atom are indicated with gray dashed lines.

The SFX structure of the H_2_O_2_ soaked DtpB revealed a new electron density peak on the distal side of the heme‐Fe in each of the six DtpB monomers that make up the crystallographic asymmetric unit (Figure 3 A). An oxygen atom at 1.65±0.11 Å distance from the heme‐Fe has been modelled in monomer A (Figure 3 A), with the mean distance averaged over all six monomers being 1.82 Å (Table S3). A composite compound I X‐ray structure at 100 K (Tables S1 and S2) was determined in order to assess temperature effects on structure and further confirmed the presence of a short Fe−O bond of 1.65±0.17 Å in monomer A, with the mean distance in all six monomers being 1.76 Å (Figure S5 and Table S3). Fe−O bond lengths for compound I and II from several structures of nonmammalian peroxidases determined at 100 K are reported in Table 1, with the short Fe−O bond in DtpB most consistent with a compound I Fe^IV^=O species.

**Table 1:** Fe^IV^−O bond lengths from various structure determinations of peroxidases. For DtpB, the range of bond lengths observed for the monomers in the asymmetric unit are given.

Peroxidase (temperature [K])	Compound I Fe^IV^−O [Å]	Compound II Fe^IV^−O [Å]
DtpB (RT)	1.65–1.89	–
DtpB (100 K)	1.65–1.84	–
APX (100 K)	1.72^[3c]^	1.84^[3c]^
	–	1.88^[4b]^
HRP (100 K)	1.70^[3a]^	1.83^[3a]^
CcP (100 K)	1.62^[3c]^	1.83^[3c]^
	1.72^[3b]^	–
	1.60^[4a]^	–
	1.70^[5a]^	–

The presence of an oxo group enables a new distal site H‐bond network to be established on forming compound I. A small inwards movement of the Asn245 side chain with respect to its position in the Fe^III^ state (Figures 3 A,B) is a direct result of the side chain amide H‐bonding to the oxo group (2.78 Å mean value, Table S3). Similarly, a subtle rotation of the guanidinium group of Arg243 occurs through H‐bonding of the N^η1^ atom to the oxo group (2.82 Å mean value, Table S3; Figures 3 A,B).

Utilizing nitrogen atoms as H‐bond donors to the oxo group is a feature in the Fe^IV^=O structures of CcP and ascorbate peroxidase (APX),[^3c^, ^5a^] where an indole ring N^*ϵ*^ atom of a Trp and a N^*ϵ*^ atom of the distal Arg (as opposed to the N^η^ atom in DtpB) act as donors. For the N^*ϵ*^ atom of the Arg to H‐bond to the oxo in CcP and APX its side chain must adopt an “in” conformation as opposed to the “out” conformation observed in the Fe^III^ structure.[^3c^, ^22^] No Arg side chain switching is observed for DtpB, which is fixed in position by H‐bonds from the O1 atom of heme propionate‐7 to the N^η^ atoms in both the Fe^III^ and Fe^IV^=O states. These H‐bonding interactions together with the porphyrin‐π cation radical withdrawing electron density from the oxo group, will make the protonation of the Fe^IV^=O difficult. Given that protonation is a precursor for the oxo species to act as a potent oxidant, the combination of Arg and Asn as H‐bonding donors must tune the p*K*_a_ of the oxo in DtpB to result in a stable, unreactive compound I. Compound I *t*_1/2_ times for the bacterial DyPs from *Rhodococuus jostii* RHA1^[17a]^ and *Bacillus subtilis*,^[23]^ which both possess a distal Asn, are reported to be 540 s and 3.2 h, respectively. Therefore, the SFX structure of the Fe^IV^=O DtpB illustrates that the interaction of the Asn with the oxo group must, amongst other factors, serve to stabilize compound I. In contrast, Asp152 is not H‐bonded to the oxo group, with the O^δ^ atoms being >4.4 Å apart (Table S3). In CcP the catalytic distal His is not within H‐bonding distance to the oxo group, and instead the distal H_2_O molecule facilitates proton movement, bridging between the oxo group and the His N^*ϵ*^ atom.^[5a]^ Thus the absence of H_2_O in the distal site of either the Fe^III^ or Fe^IV^=O DtpB structures provides a strong indication that a H_2_O‐Asp mediated mechanism of O−O bond cleavage as described in DtpA,^[3e]^ cannot occur and thus an alternative mechanism must be sought.

**Figure 4:**
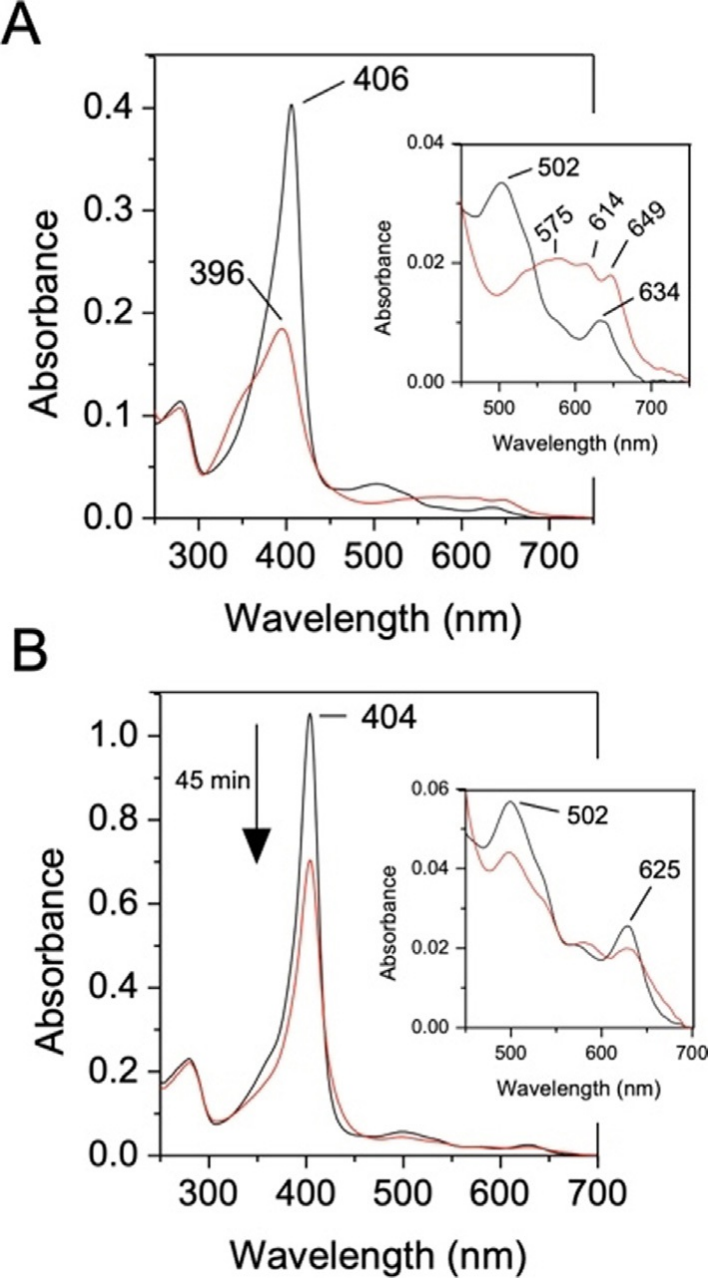
Electronic absorption spectra of A) the D152A and B) the R243A variants with wavelength absorbance maxima (nm) indicated (pH 5.0). Insets: magnified Q‐band region. On addition of one molar equivalent H_2_O_2_, the Fe^III^‐D152A variant (black) instantly forms compound I (red), whereas the Fe^III^‐R243A variant (black) slowly decays, with the spectrum recorded after 45 min illustrated (red).

We suggest that a “dry” distal site arises through the presence of an Asn, impeding the formation of the H_2_O‐Asp unit, which in DtpA, is required for heterolysis of H_2_O_2_ and rate enhancement of compound I formation.^[3e]^ In a “dry” site, we propose that the distal Arg is a potential alternative to facilitate proton movement to form compound I. To test this structure‐based hypothesis, we created the D152A and R243A variants of DtpB. Addition of one molar‐equivalent of H_2_O_2_ to the Fe^III^‐D152A variant resulted in the instantaneous formation of an electronic absorbance spectrum that in analogy with wild‐type (WT) DtpB can be assigned as compound I (Figure 4 A). From stopped‐flow kinetics a linear dependence of pseudo‐first order rate constants on [H_2_O_2_] is observed (Figure S6) yielding *k*_1_=1.7±0.1×10^5^ M^−1^ s^−1^ (25 °C). Thus, the D152A variant displays kinetic and spectral properties that are equivalent to those of WT DtpB in compound I formation. In contrast, addition of one molar equivalent of H_2_O_2_ to the Fe^III^‐R243A variant does not instantaneously lead to compound I formation. Instead the electronic absorbance spectrum reveals a slow bleaching over time of the Fe^III^ state (Figure 4 B). Global analysis of the spectral transitions on mixing the R243A variant with H_2_O_2_ concentrations ranging between 250–2500 μM using stopped‐flow, revealed that compound I slowly formed, hence the bleaching observed, and this, unlike the WT, decayed over many minutes to re‐form the Fe^III^ species. Thus, removal of the distal Arg slows compound I formation and renders it unstable. Slow acquisition of protons from the heme environment and electrons from the protein (auto‐reduction) leads to the slow re‐formation of the Fe^III^ form. The R243A variant therefore exhibits a more typical peroxidase catalytic cycle but executes this very slowly. Thus we propose that DyPs possessing a distal heme Asn will use Arg instead of Asp to facilitate compound I formation, as also evidenced from an earlier study.^[17a]^

**Figure 5:**
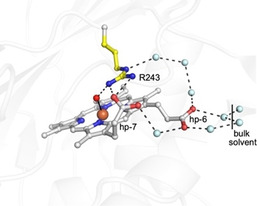
Water channels connecting the Fe^IV^=O to the bulk solvent via the distal Arg243 and the heme propionates in the SFX structure. Waters (cyan spheres) are positioned within H‐bonding range of each other (≈2.8 Å dashed lines) and may function as proton transfer pathways.

Despite the guanidinium group of Arg having a p*K*_a_ of 13.8^[24]^ and therefore protonated at neutral pH, several enzymes,^[25]^ including a metalloenzyme,^[26]^ are reported to use Arg as a catalytic base. Furthermore, several neutron structures provide evidence for the existence of neutral Arg (guanidine) side chains.^[27]^ Pertinent to the present study is a recent report highlighting the existence of a neutral distal Arg in APX when complexed to its substrate, ascorbate.^[28]^ Compound I of APX abstracts an electron and a proton from ascorbate in a concerted manner (proton coupled electron transfer; PCET) to form compound II, which then also undergoes a PCET step with an ascorbate molecule to form the Fe^III^‐heme and release of H_2_O. The distal Arg in APX has been implicated together with a distal H_2_O molecule and the heme propionates to be part of a proton delivery pathway that would require switching between the neutral and charged states.^[28]^ We note that a continuous H‐bond network from bulk solvent to the oxo group involving H_2_O channels, both heme propionates and the distal Arg243 exists in DtpB (Figure 5). Thus a case for a proton transfer pathway to the oxo group can be made, implicating the requirement for Arg243 to deprotonate. A common structural motif amongst enzymes that have Arg as an acid/base catalyst is that the guanidinium group is adjacent to a carboxylate group and solvent accessible.^[25b]^ In all DyP structures, the guanidinium group of the distal Arg is buried and H‐bonded to the carboxylate group of the inwardly facing heme propionate‐7. Furthermore, the guanidinium group is solvent accessible and thus can be considered to bear the hallmarks for a catalytic role. Chemically, it is possible for the neutral guanidine moiety to adopt five tautomeric forms in solution each corresponding to the loss of one of the five nitrogen bonded protons. These tautomers exist in a pH‐independent equilibrium and rapidly interconvert via bond rotations and/or proton transfer, with the observed p*K*_a_ the sum of the five microscopic acid dissociation constants contributed by each tautomer.^[29]^ Interactions that can “twist” the planar guanidinium group (charged) to nonplanar (neutral) may perturb the p*K*_a_.^[25b]^ In this respect the carboxylate groups of the propionate‐7 may limit the access to H‐bonds thereby forcing the NH_2_ groups into a nonplanar conformation. Thus the inwardly pointing propionate group serves to impose a steric constraint to modulate the Arg p*K*_a_. In doing so it is conceivable that only a fraction of the active enzyme will exist at any one time, but providing this fraction is kinetically competent it will be sufficient to drive compound I formation.

## Conclusion

Using SFX we have determined the pristine structures of two catalytic states of a DyP at ambient temperature. The compound I structure of DtpB is the first directly determined for a peroxidase carrying a porphyrin π‐cation radial, with the spectroscopic data and the Fe−O bond length from structures both in keeping with an Fe^IV^=O species. These SFX structures of DtpB offer the first structural insight into genuine differences between two bacterial DyP subfamilies (A vs. B). Only by unambiguously determining pristine structures of heme peroxidases can hypotheses regarding mechanisms begin to be tested, avoiding the ambiguities resulting from heme reduction that have complicated the early mechanistic studies of DyPs. In this respect SFX approaches are well suited, but access to XFEL beamlines and sample delivery, especially for detecting more short‐lived compound I and compound II states, create challenges. For DtpB the long‐lived compound I species has proved advantageous and, through revealing the “dry” nature of the distal heme site that results from the presence of an Asn, we offer a structural framework for how DyPs can modulate the distal Asp/Arg couple to facilitate proton transfer and the rate enhancement of H_2_O_2_ heterolysis.

## Conflict of interest

The authors declare no conflict of interest.

## Supporting information

As a service to our authors and readers, this journal provides supporting information supplied by the authors. Such materials are peer reviewed and may be re‐organized for online delivery, but are not copy‐edited or typeset. Technical support issues arising from supporting information (other than missing files) should be addressed to the authors.

Supplementary
