# Supplementary material for: Serial Femtosecond Zero Dose Crystallography Captures a Water‐Free Distal Heme Site in a Dye‐Decolorising Peroxidase to Reveal a Catalytic Role for an Arginine in FeIV=O Formation
Source: Angew Chem Int Ed Engl. 2020 Sep 23;59(48):21656–62. doi: 10.1002/anie.202008622 (PMC7756461; doi:10.1002/anie.202008622)
Supplement: Supplementary file 1 — Supplementary [file ANIE-59-21656-s001.pdf]

Angewandte Chemie
Eine Zeitschrift der Gesellschaft Deutscher Chemiker

Supporting Information

Serial Femtosecond Zero Dose Crystallography Captures a Water-Free Distal Heme Site in a Dye-Decolorising Peroxidase to Reveal a Catalytic Role for an Arginine in Fe^{IV}=O Formation

Marina Lučić, Dimitri A. Svistunenko, Michael T. Wilson, Amanda K. Chaplin, Bradley Davy, Ali Ebrahim, Danny Axford, Takehiko Tosha, Hiroshi Sugimoto, Shigeki Owada, Florian S. N. Dworkowski, Ivo Tews, Robin L. Owen, Michael A. Hough, and Jonathan A. R. Worrall**

anie_202008622_sm_miscellaneous_information.pdf

EXPERIMENTAL

Cloning of DtpB and construction of a plasmid for over-expression in Escherichia coli

The gene encoding for DtpB in *Streptomyces lividans* 1326 (SLI_7409) consists of 948 nucleotides and encodes for a protein of 316 amino acids. Genomic DNA was used together with the following forward and reverse primers with flanking NdeI and Hind III restriction sites (underlined): F-5'-CTGCCATATGATGGGCGGAGAAGTCGAGGAACC-3' and R-5'-CTTAAGCTTTCAGGGCCGAGCGGAGAGGTCCTC-3' to amplify *dtpB* by PCR. The resulting PCR product was ligated into a pET28a (Kan^r) vector (Novagene), to create an N-terminal His_6-tag construct (pET28*dtpB*). Several clones underwent DNA sequencing (Source Bioscience) to the confirm the expected sequence.

Over-expression and purification of DtpB

The pET28*dtpB* (Kan^r) vector was transformed into *E. coli* BL21 (DE3) cells. Overnight precultures (low salt LB medium; Melford) were successively used to inoculate 1.4 L of high salt LB medium (10 g tryptone, 10 g sodium chloride, 5 g yeast extract per litre) with 50 mg ml^{-1} kanamycin and grown at 37°C, 180 rpm. At an OD_{600} of 1.2, 5-aminolaevulinic acid (0.25 mM final concentration) and iron citrate (100 μM final concentration) were added consecutively for their use as a haem-precursor and iron supplement. Cultures were then induced by adding isopropyl β-D-thiogalactopyranoside (IPTG; Melford) to a final concentration of 0.5 mM and carbon monoxide (CO) gas bubbled through the culture for 20-30 s. Flasks were then sealed and incubated for a further 18 h at 30 °C and 100 rpm. Cells were harvested via centrifugation (10,000 g, 10 min, 4 °C) and the cell pellet resuspended in 50 mM Tris/HCl, 500 mM NaCl (Fisher) and 20 mM imidazole (Sigma) with the pH adjusted to 8 (Buffer A). The resuspended cell suspension was lysed using an EmulsiFlex-C5 cell disrupter (Avestin) followed by centrifugation (22,000 g, 30 min, 4 °C). The clarified supernatant was loaded onto a 5-ml nickel-nitrilotriacetic acid-Sepharose column (GE Healthcare) equilibrated with Buffer A and eluted by a linear imidazole gradient using Buffer B (Buffer A with 500 mM imidazole). The peak eluting at ~ 50 % Buffer B contained DtpB and was pooled and concentrated using a centricon (VivaSpin) with a 10 kDa cut-off at 4 °C followed by loading onto a PD-10 column (Generon) equilibrated in 20 mM NaPi, 100 mM NaCl, pH 7 to remove imidazole. The eluant was further concentrated for application to a S200 Sephadex column (GE Healthcare) equilibrated with 20 mM NaPi, 100 mM NaCl, pH 7. A major peak eluted at

~ 55 ml consistent with a monomer species with fractions assessed by SDS-PAGE then concentrated and stored at - 20 °C.

Site directed mutagenesis

Single amino acid substitutions were created using the QuikChange protocol (Stratagene). The pET28*dtpB* was used as template and the following forward and reverse primers (Sigma) were used to create mutations that would result in the D152A and R243A mutants, respectively; D152A-F 5'-CTCGGTTTCGTCGCCGGCACGGAGAAC-3', D152A-R 5'-GTTCTCCGTGCCGGCGACGAAACCGAG-3', R243A-F 5'-CGATCTGGAGATCCTGGCGGACAACATGCCCTTC-3', R243A-R 5'-GAAGGGCATGTTGTCCGCCAGGATCTCCAGATCG-3'. A PCR mix consisting of the respective primers (75 ng/µL), the template (15 ng/µL), 10 mM dNTPs (Fermentas), *Pyrococcus furiosus* (*Pfu*) Turbo polymerase (Agilent), 10 x *Pfu* buffer (Agilent), 8% DMSO and deionised H_2O was prepared and subjected to the following PCR cycle; 95 °C for 3 min; 16 cycles of 95 °C for 1 min, 62 °C for 1 min (D152A) or 54 °C for 1 min (R243A) and 72 °C for 8 min; 72 °C for 15 min. Clones were corroborated for the presence of the desired mutation by DNA sequencing (Eurofins).

Sample preparation

DtpB was exchanged into a desired buffer using a PD-10 column (Generon) and concentrated using centrifugal ultrafiltration devices (Vivaspin GE Healthcare). Enzyme concentration was determined by UV-visible spectroscopy (Varian Cary 60 UV-visible spectrophotometer) using an extinction coefficient (ε) at 280 nm of 18,575 M^{-1} cm^{-1}. H_2O_2 solutions (Sigma-Aldrich) were prepared from a stock with the final concentration determined spectrophotometrically using an ε = 43.6 M^{-1} cm^{-1} at 240 nm.[1]

Stopped-flow absorbance spectroscopy

Transient kinetics of the interaction of H_2O_2 with ferric DtpB and variants was performed using a SX20 stopped-flow spectrophotometer (Applied Photophysics, UK) equipped with a diode-array multi-wavelength unit and thermostatted to 25 °C. DtpB solutions (10 µM before mixing) were prepared in 50 mM sodium acetate, 150 mM NaCl, pH 5 and mixed with a series of H_2O_2 concentrations (ranging from 10 – 600 µM for wild-type; 40 – 200 µM for D152A; 500 – 5000 µM for R243A; before mixing). The overall spectral transitions were monitored and fitted to

models in the Pro-K software (Applied Photophysics, UK) to yield pseudo-first order rate constants for Compound I (k_{obs1}) formation.

EPR spectroscopy and simulation

Wilmad SQ EPR tubes (Wilmad Glass, Buena, NJ) with OD = 4.05 ± 0.07 mm and ID = 3.12 ± 0.04 mm (mean ± range) were used. Samples frozen in a set of these tubes yielded very similar intensities of EPR signals; with only ~1-3% random error. All EPR spectra were measured on a Bruker EMX EPR spectrometer (X-band) at a modulation frequency of 100 kHz. A Bruker resonator ER 4122 (SP9703) and an Oxford Instruments liquid helium system were used to measure the low-temperature (10 K) EPR spectra. EPR spectra of a blank sample (frozen water) measured at the same set of instrumental conditions were subtracted from the DtpB spectra to eliminate the background baseline EPR signal. Spectra deconvolution into two components and measurements of the intensities of these components in the time dependence set of samples were performed by using the procedure of spectra subtraction with variable coefficient.[2] Quantitative estimates of the concentrations of the paramagnetic centres were performed by comparison of the second integrals simulated EPR signals with a reference to known total concentration of the ferric haem in the sample. Simulation was performed by WinEPR SimFonia (Bruker).

Preparation of time series for EPR spectroscopy

A time series of DtpB samples following activation by H_2O_2 was created in two ways. The first procedure required the addition of a stock solution of H_2O_2 (~10 mM) to a DtpB sample (40 μM) to give a 1:10 ratio (DtpB:H_2O_2), from which an aliquot was drawn and frozen in methanol kept on dry ice (~195 K). This method provided the freezing time (*i.e.* the reaction time) from 11 s and upwards. The second procedure to enable sub 10 s sample preparation required the stock H_2O_2 solution to be inserted into plastic tubing connected to the syringe used to draw the DtpB sample from the EPR tube, which was subsequently loaded back to the EPR tube and frozen, providing freezing times from 4 s.

Crystallisation and H_2O_2 soaking of DtpB crystals

Various crystal sizes of ferric DtpB were grown under batch conditions and were used accordingly for SFX or conventional X-ray studies. Microcrystals for SFX were grown by mixing in microfuge tubes a 1:1 v/v ratio of a solution containing 6-10 mg/ml DtpB in 50 mM

sodium acetate, 150 mM NaCl pH 5 with a precipitant solution consisting of 150 mM $MgCl_2$, 150 mM HEPES, 20% PEG 4000 with the pH adjusted to 7.5, to give a final volume of 200 µl. Crystals (~10 x 8 µm) grew at room temperature within a 24 - 48 h period and displayed no signs of deterioration over several months. Larger crystals (~400 x 100 µm) for conventional cryo-cooled X-ray crystallography were obtained by mixing 1:1 v/v solutions of 6-10 mg/ml DtpB in 50 mM sodium acetate, 150 mM NaCl pH 5 with a precipitant solution consisting of 100 mM $MgCl_2$, 100 mM HEPES pH 7.5, 16% PEG 4000. Crystals grew within 48 h and were stable for many months. To generate compound I in the microcrystals a stock solution of H_2O_2 was added to a 200 µl microcrystal containing suspension to a final concentration of 600 µM. Larger crystals for cryo-crystallography were removed from the batch solution by pipette and transferred to a drop consisting of H_2O_2 (final concentration 600 µM), 20% w/v glycerol (cryo-protectant) in mother liquor on a siliconized cover slip for 30 s before flash-cooling.

Microspectrophotometry and X-ray data collection at 100 K

Crystals of ferric and H_2O_2 soaked DtpB were cryo-protected in mother liquor containing 20% w/v glycerol and flash-cooled in liquid nitrogen. X-ray diffraction and single crystal spectroscopic data at 100 K were collected at the Swiss Light Source (SLS) beamline X10SA. The MS3 on-axis microspectrophotometer[3] was used to measure absorbance spectra of ferric DtpB and H_2O_2 soaked DtpB crystals in the range of 450 to 700 nm. Each spectrum was the result of 50 accumulations of 100 ms exposures. Spectra were measured prior to and following X-ray data collection and a dose limit was selected such that minimal changes occurred to the spectrum of the ferric and ferryl forms during data collection from each crystal. A multi-crystal approach was performed to obtain a complete low-dose composite dataset for the ferric and ferryl DtpB structures. A total of 21 (ferric DtpB) and 13 (ferryl DtpB) spectroscopically-validated diffraction data wedges were merged using the in house go2gether.com script in the XDS package.[4] X-ray absorbed doses were estimated using Raddose-3D.[5] To reflect the beam profile used, a weighted average of doses calculated for top hat and Gaussian profiles was calculated and doses were estimated for the range of crystal dimensions used.

Serial femtosecond X-ray crystallography (SFX)

Silicon fixed-target chips with either 12 or 14 µM apertures at their narrowest opening and a nominal capacity of 25,600 were loaded with 200 µl of microcrystal suspension within a humidity enclosure and sealed between two layers of 6 µm thick Mylar. We have described

this approach in more detail previously.[6] SFX data were measured at SACLA beamline BL2 EH3 using an X-ray energy of 11 keV, a pulse length of 10 fs, beam size 1.6 x 1.6 μm and a repetition rate of 30 Hz. Chips were translated within the interval between X-ray pulses, ensuring that the chip had stopped at the centre of each crystal position (the centre of the aperture) and was exposed only once to X-rays, before moving to the next pulse interval. Data was typically collected from all 25,600 positions on a chip in < 15 min using the SACLA MPCCD detector.[7] SFX data were processed using the CHEETAH pipeline[8] and CrystFEL[9] with scaling and merging using the *Partialator* program.

DtpB structure determination and refinement

The ferric SFX structure was solved by molecular replacement using MrBUMP[10] and BUCCANEER.[11] The search model identified by MrBUMP for molecular replacement was 3QNR.[12] Initial refinement of the structure was carried out in PHENIX,[13] using torsion-angle simulated annealing to eliminate model bias and completed using Refmac5[14] in the CCP4i2 suite.[15] Model building between refinement cycles was performed in Coot.[16] Riding hydrogen atoms were added during refinement. The ferryl DtpB SFX structure ferric and ferryl 100 K structures were determined from a starting model of the ferric SFX structure, with the same simulated annealing and refinement procedures used. No restraints were placed on the Fe-$N^{\varepsilon 2}$His and Fe-O distances. All structures were validated using the Molprobity server,[17] the JCSG Quality Control Server and tools within Coot.[16] Bond length error estimates were obtained using atomic diffraction precision index values obtained from the Online_DPI server.[18] A summary of data collection and refinement statistics are given in Table S1 and Table S2, respectively.

Table S1: SFX (ambient temperature) and composite (100 K) X-ray crystallography data processing for DtpB in space group $P2_12_12_1$. Values in parenthesis refer to the outermost resolution shell. The effective absorbed X-ray dose for the SFX structures is assigned as zero due to the femtosecond duration of the X-ray pulse. As is standard practice for SFX data,[19] the metrics R_{split} and $CC_{1/2}$ are used to assess data quality and resolution limit in place of conventional crystallographic metrics such as R_{merge} or I/sd(I).

	**SFX ferric**	**SFX ferryl**	**Composite ferric**	**Composite ferryl**
Chips used	4	5	-	-
Wavelength (Å)	1.13	1.13	0.8	0.8
Effective absorbed X-ray dose (kGy)	0	0	11.4 kGy	11.3 kGy
Number of crystals	26223	57909	21	13
Unit cell dimensions (Å)	86.7, 121.6, 199.0	86.3, 121.1, 198.5	85.8, 120.3, 196.0	85.4,119.9, 194.2
Resolution (Å)	13-1.85	13-1.75	49-1.99	48-1.85
Number of reflections	175001	207440	138290	159520
R_{split}	0.136 (0.678)	0.109 (0.887)	-	-
$CC_{1/2}$	0.976 (0.569)	0.984 (0.423)	0.97 (0.50)	0.95 (0.51)
R_{merge}	-	-	0.227 (1.02)	0.179 (0.567)
R_{pim}	-	-	0.088 (0.472)	0.101 (0.451)
(I)/sd(I)	-	-	7.5 (2.1)	4.4 (1.2)
Multiplicity	518 (360)	1943 (1359)	6.9 (5.0)	3.1 (1.9)
Completeness (%)	100 (100)	100 (100)	99.2 (97.1)	94.2 (79.8)
Wilson B-factor ($Å^2$)	26.9	28.4	28.2	22.2

Table 2: Refinement and validation statistics for SFX (room temperature) and composite X-ray crystallography for DtpB. Values in parenthesis refer to the outermost resolution shell.

	SFX ferric	SFX ferryl	Composite ferric	Composite ferryl
Number of reflections	174340	206723	138089	159452
Resolution (Å)	13-1.85	13-1.75	49-1.99	47.6-1.85
R_{work}	0.159	0.167	0.176	0.189
R_{free}	0.192	0.199	0.218	0.232
RMSD bond lengths (Å)	0.012	0.011	0.009	0.009
RMSD bond angles (°)	1.7	1.6	1.6	1.6
Solvent atoms	810	880	1142	1195
ESU based on R_{free} (Å)	0.106	0.099	0.152	0.142
ESU based on ML (Å)	0.088	0.078	0.116	0.120
Ramachandran most favoured (%)	97	97	97	97
PDB accession code	6YRJ	6YRD	6YRC	6YR4

RESULTS

Table S3: Coordinate and hydrogen bond lengths for the haem site in the various DtpB structures. Distances for the distal triad of Asp152, Arg243 and Asn245 to the oxo in the ferryl structures are measured from the closest O^{δ} atom of Asp152, the $N^{\eta 1}$ atom of Arg243 and the side-chain amino group of Asn245. The mean distance for the six monomers in the crystallographic asymmetric unit is reported together with the associated standard deviation in parentheses.

Chain	SACLA ferric	SLS ferric	SACLA ferryl					SLS ferryl				
	Fe-N^{ε}His (Å)	Fe-N^{ε}His (Å)	Fe-N^{ε}His (Å)	Fe=O (Å)	Fe=O-Asp (Å)	Fe=O-Arg (Å)	Fe=O-Asn (Å)	Fe-N^{ε}His (Å)	Fe=O (Å)	Fe=O-Asp (Å)	Fe=O-Arg (Å)	Fe=O-Asn (Å)
A	2.05	2.09	2.08	1.65	4.39	2.89	2.96	2.09	1.65	4.33	2.94	2.84
B	2.10	2.10	2.13	1.89	4.95	2.83	2.72	2.16	1.81	4.19	3.00	2.66
C	2.04	2.09	2.11	1.89	4.25	2.76	2.67	2.14	1.75	4.12	2.86	2.70
D	2.03	2.09	2.08	1.85	4.28	2.86	2.78	2.16	1.83	4.21	2.69	2.68
E	2.02	2.01	2.06	1.83	4.43	2.79	2.77	2.10	1.84	4.16	2.66	2.76
F	2.00	2.06	2.12	1.80	4.28	2.79	2.80	2.07	1.70	4.37	2.77	2.98
Mean distance	2.04 (0.03)	2.07 (0.03)	2.10 (0.02)	1.82 (0.08)	4.43 (0.24)	2.82 (0.04)	2.78 (0.09)	2.12 (0.04)	1.76 (0.07)	4.23 (0.09)	2.82 (0.12)	2.77 (0.11)

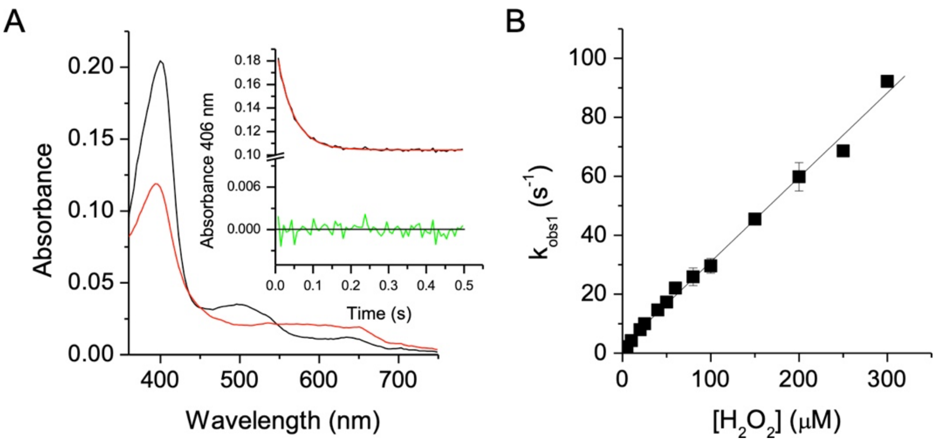

Figure S1: Detection of compound I in DtpB using stopped-flow absorption spectroscopy (pH 5.0 and 25 °C). A) Spectra obtained from global fitting of the observed spectral transitions upon mixing H_2O_2 (60 μM) with Fe^{III}-DtpB (5 μM) according to the model a → b (a = ferric b = compound I). Inset shows a kinetic trace at 406 nm along with the fit (red) and residual (green) to the model. B) Pseudo first-order rate constants (k_{obs1}) plotted against [H_2O_2]. The data points are fitted to a linear function to obtain a second-order rate constant.

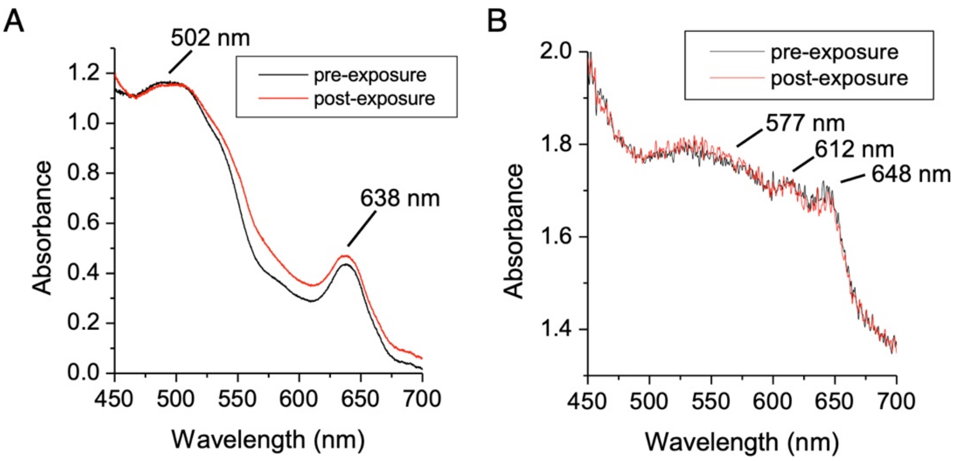

Figure S2: Electronic absorption spectra of DtpB crystals. The α/β band region recorded at 100 K using the on-axis UV-vis microspectrophotometer MS3[3] at beamline X10SA of the Swiss Light Source for a crystal of A) Fe^{III}-DtpB and B) Fe^{IV}=O compound I. The ferryl species was formed following the addition of H_2O_2 (final concentration 600 μM) to a drop containing Fe^{III}-DtpB crystals. The spectra of pre and post X-ray exposure following collection of a data wedge (10° ferric, 8° ferryl) are shown.

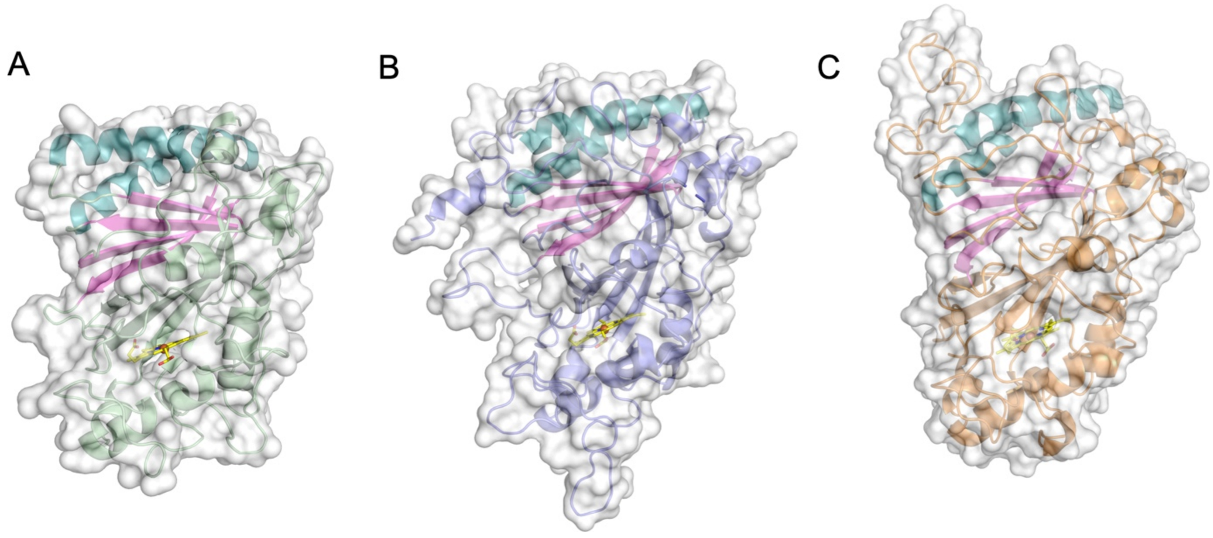

Figure S3: Tertiary structure of DtpB and comparison with DtpA and DtpAa. Cartoon of the overall structure and surface representation of A) SFX structure at ambient temperature of Fe^{III}-DtpB, B) cryo-cooled (100 K) Fe^{III}-DtpA (PDB code 6GZW) and C) cryo-cooled (100 K) Fe^{III}-DtpAa (PDB code 6TB8). The fold for one of the ferredoxin-like domains in each structure is highlighted and the haem is shown in sticks.

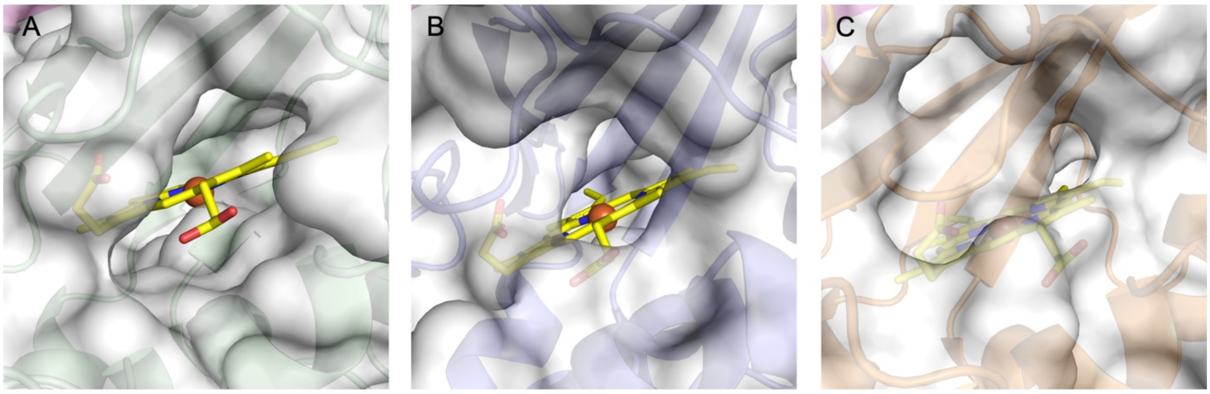

Figure S4: Depiction of haem pocket surface opening in A) Fe^{III}-DtpB, B) Fe^{III}-DtpA and C) Fe^{III}-DtpAa. The haem solvent accessible surface area ($Å^2$) for each structure reported in the main text was calculated using NACCESS v 2.1.1.[20]

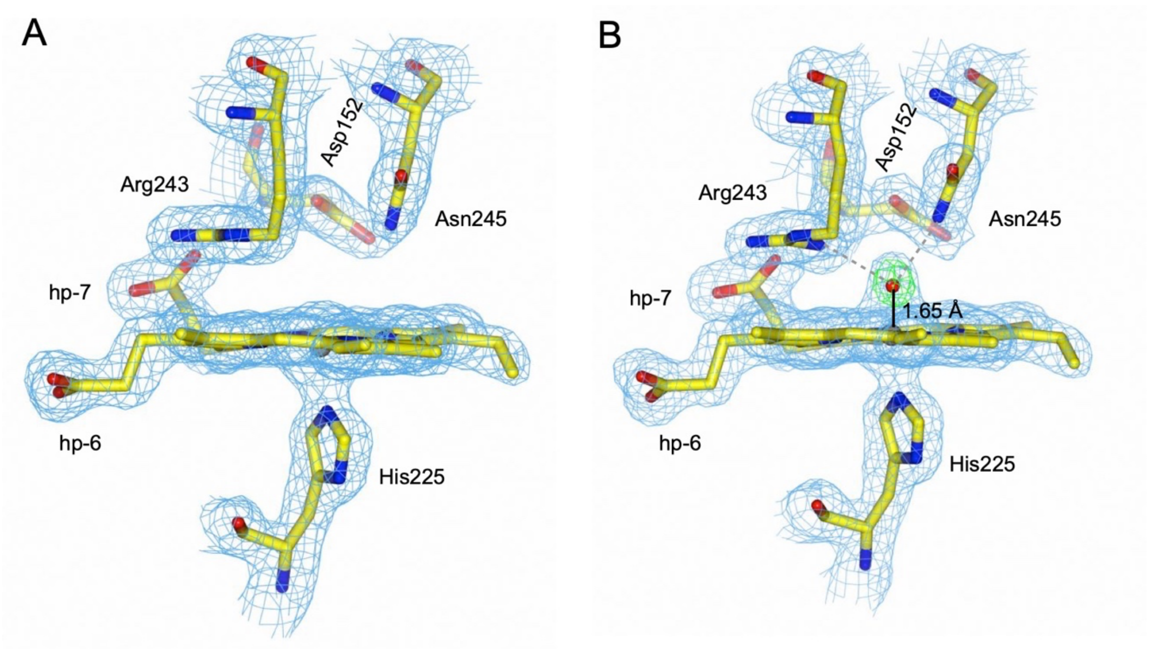

Figure S5: Distal pockets of the Fe^{III} and Fe^{IV}=O states of DtpB determined at 100 K. $2F_o$-F_c electron density maps (blue) contoured at 1.4 σ for the Fe^{III} (A) and Fe^{IV}=O (B) oxidation states. In (B) the F_o-F_c map (green) is also shown, contoured at ± 7.5 σ and was calculated after OMIT refinement omitting the oxygen atom (red sphere). The Fe to O coordination bond is shown as a solid black line and H-bond interactions are indicated with grey dashed lines.

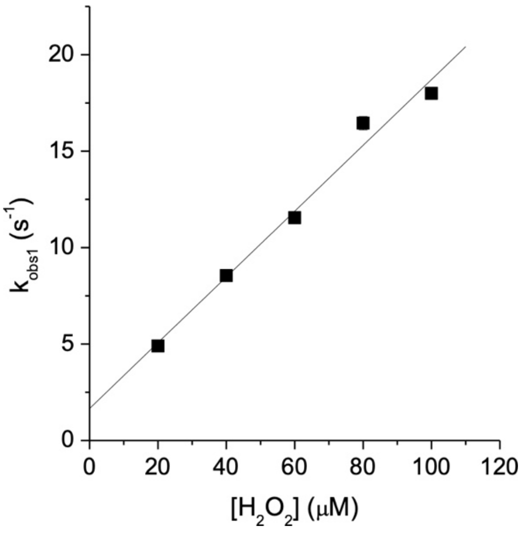

Figure S6: Pseudo first-order rate constants (k_{obs1}) obtained at 25 °C and pH 5.0, plotted against [H_2O_2] for the D152A variant of DtpB (5 μM). The data points are fitted to a linear function to obtain a second-order rate constant (k_1) for compound I formation.

REFERENCES

[1] R. F. Beers, Jr., I. W. Sizer, *J. Biol. Chem.* **1952**, *195*, 133-140.

[2] D. A. Svistunenko, N. Davies, D. Brealey, M. Singer, C. E. Cooper, *Biochim. Biophys. Acta* **2006**, *1757*, 262-272.

[3] M. R. Fuchs, C. Pradervand, V. Thominet, R. Schneider, E. Panepucci, M. Grunder, J. Gabadinho, F. S. Dworkowski, T. Tomizaki, J. Schneider, A. Mayer, A. Curtin, V. Olieric, U. Frommherz, G. Kotrle, J. Welte, X. Wang, S. Maag, C. Schulze-Briese, M. Wang, *J. Synchrotron Radiat.* **2014**, *21*, 340-351.

[4] W. Kabsch, *Acta Crystallogr. Sect. D* **2010**, *66*, 125-132.

[5] O. B. Zeldin, M. Gerstel, E. F. Garman, *J. Appl. Crystallogr.* **2013**, *46*, 1225-1230.

[6] T. Moreno-Chicano, A. Ebrahim, D. Axford, M. V. Appleby, J. H. Beale, A. K. Chaplin, H. M. E. Duyvesteyn, R. A. Ghiladi, S. Owada, D. A. Sherrell, R. W. Strange, H. Sugimoto, K. Tono, J. A. R. Worrall, R. L. Owen, M. A. Hough, *IUCrJ* **2019**, *6*, 1074-1085.

[7] T. Kameshima, S. Ono, T. Kudo, K. Ozaki, Y. Kirihara, K. Kobayashi, Y. Inubushi, M. Yabashi, T. Horigome, A. Holland, K. Holland, D. Burt, H. Murao, T. Hatsui, *Rev. Sci. Instrum.* **2014**, *85*, 033110.

[8] A. Barty, R. A. Kirian, F. R. Maia, M. Hantke, C. H. Yoon, T. A. White, H. Chapman, *J. Appl. Crystallogr.* **2014**, *47*, 1118-1131.

[9] T. A. White, V. Mariani, W. Brehm, O. Yefanov, A. Barty, K. R. Beyerlein, F. Chervinskii, L. Galli, C. Gati, T. Nakane, A. Tolstikova, K. Yamashita, C. H. Yoon, K. Diederichs, H. N. Chapman, *J. Appl. Crystallogr.* **2016**, *49*, 680-689.

[10] R. M. Keegan, M. D. Winn, *Acta Crystallogr. Sect. D* **2008**, *64*, 119-124.

[11] K. Cowtan, *Acta Crystallogr. Sect. D* **2006**, *62*, 1002-1011.

[12] R. Singh, J. C. Grigg, Z. Armstrong, M. E. Murphy, L. D. Eltis, *J. Biol. Chem.* **2012**, *287*, 10623-10630.

[13] D. Liebschner, P. V. Afonine, M. L. Baker, G. Bunkoczi, V. B. Chen, T. I. Croll, B. Hintze, L. W. Hung, S. Jain, A. J. McCoy, N. W. Moriarty, R. D. Oeffner, B. K. Poon, M. G. Prisant, R. J. Read, J. S. Richardson, D. C. Richardson, M. D. Sammito, O. V. Sobolev, D. H. Stockwell, T. C. Terwilliger, A. G. Urzhumtsev, L. L. Videau, C. J. Williams, P. D. Adams, *Acta Crystallogr. Sect. D* **2019**, *75*, 861-877.

[14] G. N. Murshudov, A. A. Vagin, E. J. Dodson, *Acta Crystallogr. Sect. D* **1997**, *53*, 240-255.

[15] L. Potterton, J. Agirre, C. Ballard, K. Cowtan, E. Dodson, P. R. Evans, H. T. Jenkins, R. Keegan, E. Krissinel, K. Stevenson, A. Lebedev, S. J. McNicholas, R. A. Nicholls, M. Noble, N. S. Pannu, C. Roth, G. Sheldrick, P. Skubak, J. Turkenburg, V. Uski, F. von Delft, D. Waterman, K. Wilson, M. Winn, M. Wojdyr, *Acta Crystallogr. Sect. D* **2018**, *74*, 68-84.

[16] P. Emsley, B. Lohkamp, W. G. Scott, K. Cowtan, *Acta Crystallogr. Sect. D* **2010**, *66*, 486-501.

[17] I. W. Davis, A. Leaver-Fay, V. B. Chen, J. N. Block, G. J. Kapral, X. Wang, L. W. Murray, W. B. Arendall, 3rd, J. Snoeyink, J. S. Richardson, D. C. Richardson, *Nucleic Acids Res.* **2007**, *35*, W375-383.

[18] K. S. D. Kumar, M. Gurusaran, S. N. Satheesh, P. Radha, S. Pavithra, K. P. S. T. Tharshan, J. R. Helliwell, K. Sekar, *J. Appl. Crystallogr.* **2015**, *48*, 939-942.
[19] T. A. White, R. A. Kirian, A. V. Martin, A. Aquila, K. Nass, A. Barty, H. N. Chapman, *J. Appl. Cryst.* **2012**, *45*, 335-341.
[20] S. J. Hubbard, J. M. Thornton, 'NACCESS' Computer Program, Department of Biochemistry amd Molecular Biology, University College London. ed., **1993**.
